# A two-step processing method is recommended to inactivate human sporadic M1 and V2 prions

**DOI:** 10.1099/jgv.0.002326

**Published:** 2026-09-16

**Authors:** Shirou Mohri, Yuichi Matsuura, Piero Parchi, Tetsuyuki Kitamoto

**Affiliations:** 1 Department of Neurological Science, Tohoku University Graduate School of Medicine, 2-1 Seiryou-machi, Aoba-ku, Sendai, 980-8575, Japan; 2 National Institute of Animal Health, NARO, Tsukuba, Ibaraki 305–0856, Japan; 3 Department of Health Sciences, Unit of Medical and Dental Sciences, Nagasaki University Graduate School of Biomedical Sciences, 1-12-4 Sakamoto, Nagasaki, 852-8523, Japan; 4 IRCCS Istituto delle Scienze Neurologiche di Bologna, Bologna, Italy; 5 Department of Biomedical and Neuromotor Sciences (DIBINEM), University of Bologna, Bologna, Italy

**Keywords:** decontamination of sporadic Creutzfeldt–Jakob disease (sCJD) prion, prevention of secondary infection, alkaline detergent, synergistic effects of combination procedures

## Abstract

Most human-acquired prion diseases result from infection with M1 or V2 prions from sporadic Creutzfeldt–Jakob disease. To prevent transmission via surgical instruments, sterilization methods targeting these prions are essential. In this study, stainless steel wires coated with M1 or V2 prions were used to evaluate inactivation protocols. None of the individual treatments – autoclaving, SDS or NaOH – eliminated infectivity except for autoclaving against V2 prions. Autoclaving at 134 °C revealed distinct thermal stability between M1 and V2, with M1 exhibiting greater thermal stability. Importantly, combining methods, which we herein called two-step processing methods, achieved practical inactivation: SDS treatment followed by autoclaving at 121 °C was highly effective. Commercial alkaline detergents also proved effective against M1 prion under a combination of high pH and temperature. The inability to detect infection in our model mice using our assay system demonstrates that the infectivity titre decreased to 10⁻⁸ in M1 and 10⁻⁷ in V2. These findings indicate that synergistic approaches can achieve prion inactivation under practical conditions, supporting the need for revised sterilization protocols to prevent iatrogenic transmission.

**Impact Statement**

This is a highly significant report demonstrating the direct inactivation of human prions from sporadic Creutzfeldt–Jakob disease (sCJD). Combining currently implemented on-site procedures with autoclave treatment enables it possible to inactivate human sCJD, M1 and V2 prions.

## Data Summary

The authors confirm that all supporting data, code and protocols have been provided within the article or through supplementary data files.

## Introduction

Creutzfeldt–Jakob disease (CJD) in humans has three aetiologies: sporadic CJD (sCJD), which is predominant and accounts for ~85% of cases; genetic CJD, which represents 10–15% of patients; and acquired CJD, which occurs through exposure to prions through cannibalism (Kuru), ingestion of prion-contaminated food (variant CJD, vCJD) or medical procedures (iatrogenic CJD, iCJD) [1]. More than 200 cases of acquired prion diseases have demonstrated human-to-human transmission, notably through dura mater transplantation, growth hormone therapy and Kuru [2, 3].

Molecular classification of sCJD prion strains is based on the codon 129 polymorphism and the molecular weight of proteinase K-resistant PrP fragments [4, 5], and the prion strains involved in iCJD cases are primarily sCJD-derived M1 and V2 prions [6, 7]. Although prion strain differences are known to influence sterilization efficacy [8, 9], their specific impact on the inactivation of human M1 and V2 prions remains insufficiently characterized. Consequently, developing sterilization methods that specifically target these prions is critical for ensuring safe neurosurgical practices.

Current prion detection methods, including real-time quaking-induced conversion (RT-QuIC) and protein misfolding cyclic amplification (PMCA), offer high sensitivity [10–14]. However, RT-QuIC sometimes fails to correlate with infectivity, and while PMCA is highly sensitive for detecting V2 prions, its sensitivity for detecting M1 prions is not high.

This study aims to establish effective inactivation protocols for human M1 and V2 prions adhering to surgical instruments. For this purpose, three human sCJD prion samples were used: one methionine homozygous (MM1) as a source of M1 prions and two valine homozygous (VV2) for V2 prions. To evaluate the efficacy of inactivation, stainless steel wires mimicking surgical instruments were contaminated with prions and subjected to bioassay using humanized mouse models sensitive to human prion infection.

## Methods

### Human brain homogenate and stainless wire

The brains from sCJD patients, identified as MM1 (H3) and VV2 (Ak and J51), were homogenized in a ninefold volume of PBS and cleared of debris by centrifugation at 600 ***g*** for 10 min at room temperature (20–25 °C). MM1 homogenate and wire-bound were made in adjacent areas of the frozen H3 brain, while VV2 homogenates and wire-bounds were using the same fragment of Ak or J51 brain.

Prior to coating with the sCJD prion, stainless wire segments (3 mm in length and 0.3 mm in diameter) were washed five times with a 2% Triton X-100 solution and subsequently 10 times with distilled and deionized water (DDW) to remove any residues. Ten pieces of the cleaned wire were incubated in 0.1 ml of either 10% brain homogenate or a serial dilution of it. After incubation for 30 min, all wire pieces were thoroughly dehydrated on a paper towel at 20–25 °C overnight and then stored in a tube at −80 °C until used in experiments.

### Procedure for sCJD inactivation

sCJD-coated wires underwent treatment in a gravity displacement autoclave (MC-3032L model, ALP Co., Ltd., Japan) at either 121 or 134 °C for 20 min, either as is or after soaking in 3% SDS. In the procedure involving boiling in SDS, the sCJD-coated wires were boiled in a 3% SDS solution on an induction cooker for 5 min and subsequently washed with DDW at 20–25 °C. Following boiling in 3% SDS, the secondary treatment involved autoclaving at 121 or 134 °C for 20 min. In the procedure for soaking in NaOH, the sCJD-coated wires were incubated in either 1 or 2 M NaOH solution for 60 min at 20–25 °C. Subsequently, as a secondary treatment, they were autoclaved at 121 or 134 °C for 20 min.

For the immersion treatment, a medical instrument cleaning agent, commonly used in hospitals, was employed. Alkaline cleaner S CLEAN 55α (Clean Chemical Co., Ltd.) was used for the washing of surgical instruments in the hospital to treat sCJD-coated wires. The sCJD MM1 (H3)-coated wires were soaked in the alkaline cleaner at concentrations ranging from 0.12% (pH 10.7) to 2.6% (pH 12.5). The soaking temperature and duration for the alkaline cleaners were 50 or 90 °C for 5 or 20 min.

### Bioassay

For the bioassay, previously generated humanized knock-in mice were employed. KiChM was used to detect MM1 prions due to its short incubation period and high detection sensitivity, while KiVV was used for VV2 prions (Table S1 and Fig. S1, available in the online Supplementary Material). The strains of KiChM mouse exhibit high sensitivity to sCJD MM1 transmission [15], while the KiVV strain is susceptible to sCJD VV2 transmission [6]. Wires coated with sCJD were implanted into the right cerebral hemisphere of each mouse. Mice were observed for clinical symptoms at least twice a week and were euthanized when the clinical symptoms appeared, or weakened conditions due to ageing or other factors were observed. The incubation period was defined as the interval from the date of wire implantation to euthanasia, and mean values were calculated for mice confirmed positive for sCJD transmission. For groups in which transmission was not verified, the longest observation period was recorded as ‘> days’.

Kaplan–Meier survival curves depicting the probability of survival in mice were generated using GraphPad Prism version 11.0.0 for Windows (GraphPad Software, Boston, MA, USA; https://www.graphpad.com/). An event was defined as transmission of sCJD, whereas animals without detectable transmission were censored. Overall differences among groups were assessed using the log-rank test, and pairwise comparisons were performed with adjustment for multiple testing using the Holm method to control the family-wise error rate.

To verify sCJD transmission, brains from euthanized mice were bisected along the sagittal axis; the left hemisphere was preserved in buffered formalin for histological analysis, and the right was stored at −80 °C for Western blotting analysis. Both histological analysis and Western blotting were performed as previously described [16, 17].

In general, sections from all animal models were examined by IHC (immunohistochemistry). For the PrP^Sc^-positive group identified by IHC, Western blotting was performed on a subset of cases as positive controls; as expected, all tested samples were Western blotting-positive. Western blotting was also performed on cases that were questionable or negative by IHC. Among the questionable cases, ~15% tested positive by Western blotting, and these were eventually classified as positive. All IHC-negative cases were also negative by Western blotting (Supplementary Material 1) .

## Results

**Fig. 1.**
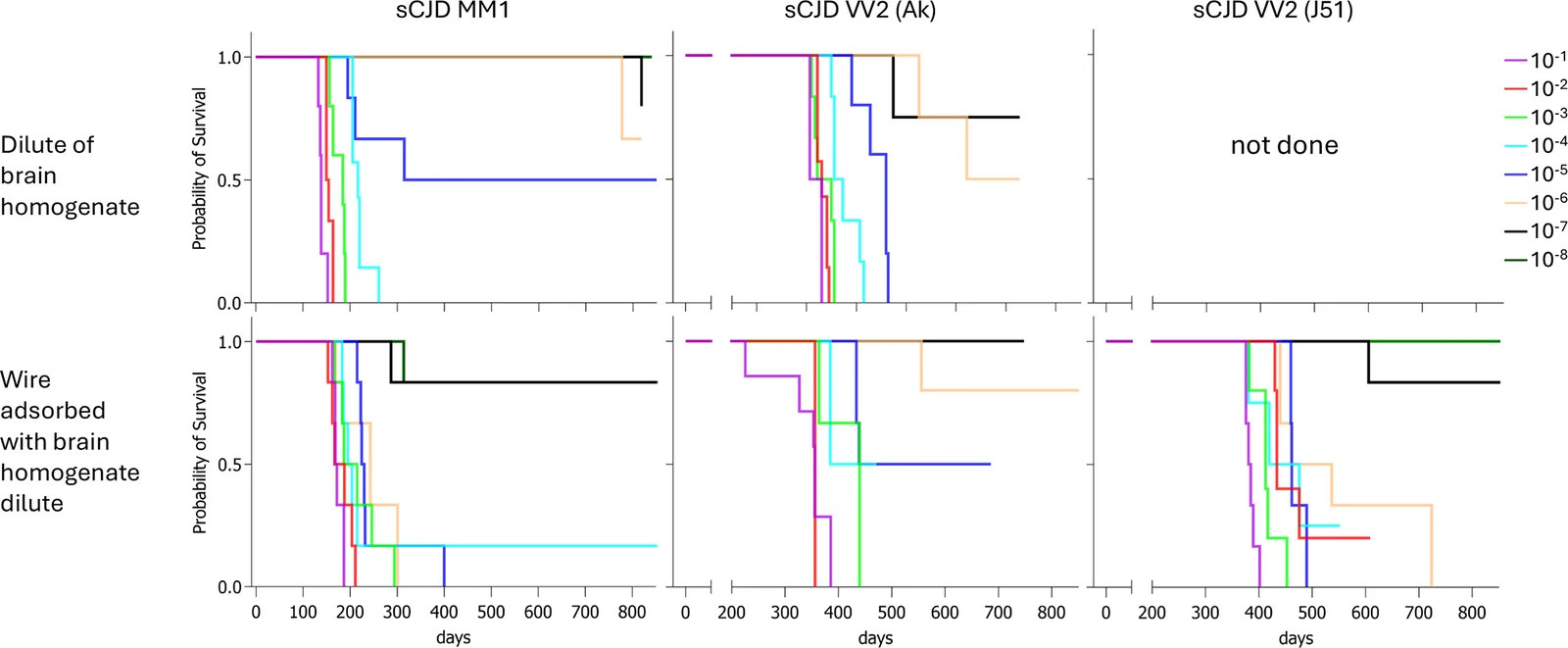
End-point titration of MM1 and VV2 (Ak and J51) prions in KiChM or KiVV mice. Tenfold serial dilutions were either intracerebrally inoculated into mice or coated onto wires followed by intracerebral implantation. Kaplan–Meier survival curves were generated to depict the probability of survival, reflecting incubation periods (days), in KiChM or Ki129VV mice. Although the overall log-rank test indicated a significant difference among groups (*P*<0.0001), no statistically significant differences were identified in pairwise comparisons after Holm correction unless differences exceeded 100-fold. The different coloured lines correspond to dilution factors and are shown on the right.

To confirm the efficiency of the stainless wire model, wires coated with serial dilutions of human brain homogenates – H3 (MM1), Ak (VV2) and J51 (VV2) – were implanted into humanized knock-in mice (KiChM or KiVV) (Table 1 and Fig. 1).

**Table 1.** Infectivity of the coated wires with serially diluted sCJD brain homogenates

	MM1 (H3) into KiChM		VV2 (AK) into KiVV		VV2 (J51) into KiVV	
Dilution rate (log_10_)	n/n_0_*	Incubation period (mean days±sd)	n/n_0_*	Incubation period (mean days±sd)	n/n_0_*	Incubation period (mean days±sd)
−1†	6/6	174±10	7/7	342±55	6/6	385±10
−2	6/6	181±24	3/3	357±0	4/6	444±22
−3	6/6	215±48	2/3	364, 440	5/5	416±25
−4	5/6	197±13	2/4	385, 385	3/4	426±48
−5	6/6	254±72	3/6	436±3	3/4	471±17
−6	6/6	240±56	1/5	556	4/5	520±121
−7	1/6	287	0/6	>748	1/6	606
−8	1/6	214	0/6	>723	0/6	>950

*Number of positive (n) and implanted mice (n_0_).

†−1 is 10% brain homogenate, and −2, −3, … show 10-fold dilution magnification each.

‘>number’ means the longest survival days of the mouse in the group.

The KiChM mice showed the highest detection rate for M1 prions with a detection limit of 10^−7^ in dilution experiments. Similarly, the KiVV mice also showed a detection limit for V2 prions of 10^−7^ dilution (Table S1). KiChM mice implanted with H3 (MM1)-coated wires exhibited stable incubation periods ranging from 174 to 215 days at 10^−1^ to 10^−3^ dilutions, with the limit of detection (LOD) determined at the 10^−8^ dilution, assuming that no mice were infected at the 10^−9^ dilution. KiVV mice implanted with Ak (VV2)- or J51 (VV2)-coated wires showed stable incubation periods at 10^−1^ to 10^−5^ dilutions, with LODs of 10^−6^ and 10^−7^ dilutions, respectively. Based on these LODs from the wire-implantation bioassay, undetected mice infected showed infectivity reduction at 10^8^ magnitudes for H3 (MM1), 10^6^ for Ak (VV2) and 10^7^ for J51 (VV2).

**Fig. 2.**
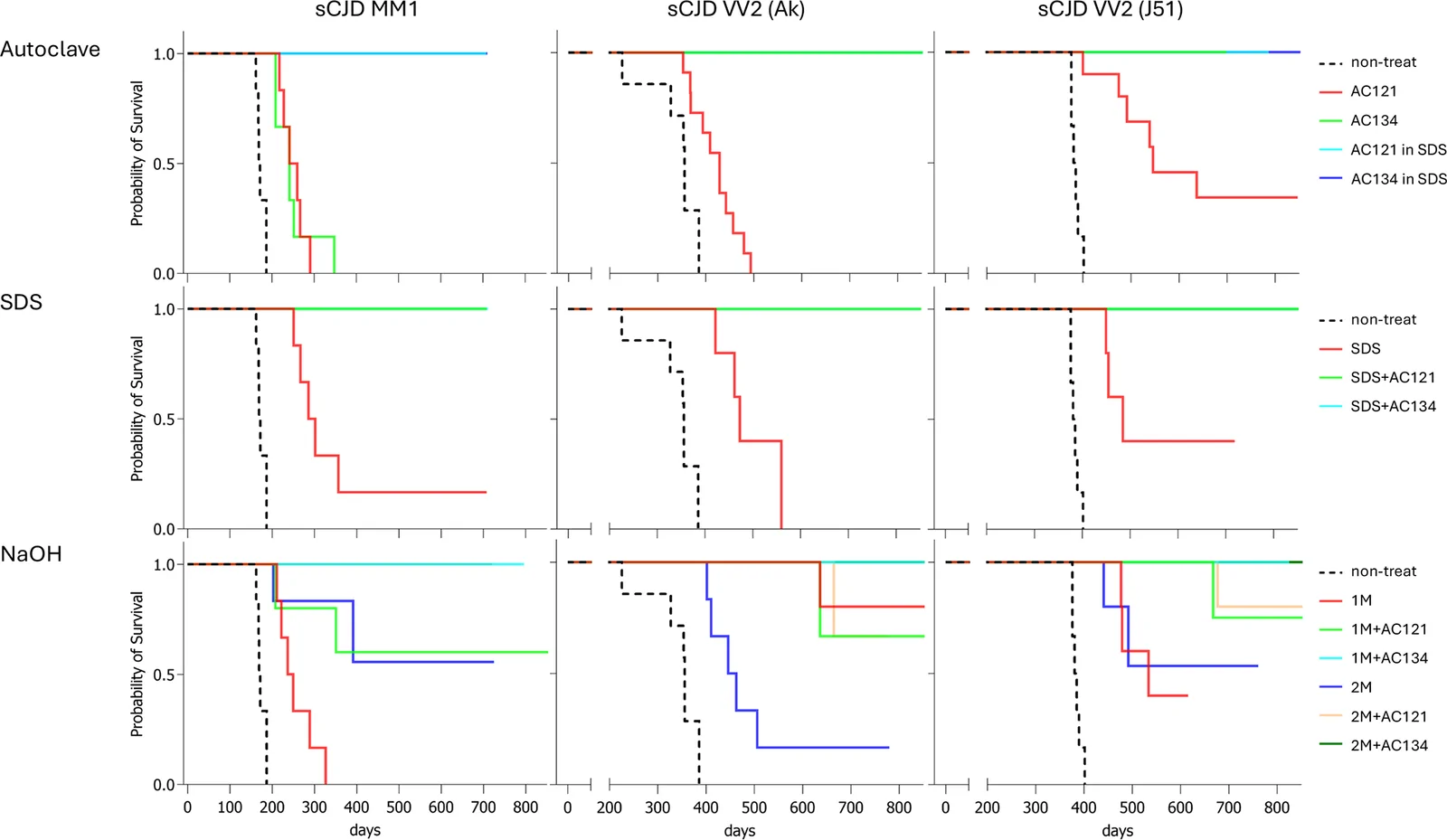
Inactivation of sCJD prions by various treatments. Kaplan–Meier survival curves were generated to depict the probability of survival, reflecting incubation periods (days), in KiChM or Ki129VV mice intracerebrally implanted with sCJD-coated wires [MM1, VV2 (Ak and J51)]. Wires were treated by autoclaving at 121 °C (AC121) or 134 °C (AC134), or by autoclaving at 121 or 134 °C in 3% SDS solution (AC121 in SDS or AC134 in SDS). Wires were also treated by boiling in 3% SDS solution followed by autoclaving at 121 or 134 °C (SDS+AC121 or SDS+AC134). In addition, wires were soaked in NaOH solution (1 or 2 M) and then autoclaved at 121 or 134 °C (1 M+AC121, 1 M+AC134, 2 M+AC121 or 2 M+AC134). Non-treated wires adsorbed with 10% brain homogenate were included as the ‘non-treat’ control. The overall log-rank test was significant (*P*<0.0001). Pairwise comparisons suggested trends towards differences; however, these did not reach statistical significance after Holm adjustment for multiple testing. The different coloured lines correspond to each treatment condition and are shown on the right.

The results of the bioassay following inactivation of sCJD-coated wires are shown in Table 2 and Fig. 2.

**Table 2.** Infectivity after the inactivation procedures on the sCJD-coated wire

	MM1 (H3)		VV2 (AK)		VV2 (J51)	
Inactivation procedure	n/n_0_*	Incubation period (mean days±sd)	n/n_0_*	Incubation period (mean days±sd)	n/n_0_*	Incubation period (mean days±sd)
**Autoclaving (AC)**						
AC 121 °C	6/6	251±27	11/11	423±44	6/10	515±80
AC 134 °C	6/6	250±51	0/5	>958	0/5	>699
AC 121 °C in 3% SDS	0/6	>708	1/9	871	0/5	>787
AC 134 °C in 3% SDS	0/5	>711	0/5	>1,056	0/5	>1,000
**Boiling in SDS**						
3% SDS	5/6	293±41	5/6	480±50	3/5	463±19
3% SDS+AC 121 °C	0/6	>708	0/5	>952	0/5	>953
3% SDS+AC 134 °C	0/6	>711	0/5	>1,083	0/5	>877
**Soaking in NaOH**						
1 M NaOH	6/6	256±44	1/5	636	3/5	497±32
1 M NaOH+AC 121 °C	2/5	280±101	1/5	636	1/5	668
1 M NaOH+AC 134 °C	0/5	>796	0/2	>792	0/6	>888
2 M NaOH	2/6	298±134	5/6	446±42	2/5	441, 492
2 M NaOH+AC 121 °C	0/6	>721	1/4	665	1/6	677
2 M NaOH+AC 134 °C	0/5	>712	0/3	>857	0/5	>962

* Number of positive (n) and implanted mice (n_0_).

† Each wire is coated with 10% brain homogenate.

‘>number’ means the longest survival days of the mouse in the group.

Autoclaving at 121 °C did not eliminate infectivity in any of the CJD strains. At 134 °C, infectivity remained in H3 (MM1) but was eliminated in Ak (VV2) and J51 (VV2), indicating a difference in heat resistance between M1 and V2 prions. When wires were autoclaved while immersed in 3% SDS, infectivity was retained in one VV2 case (AK) at 121 °C, but all prion strains, including H3 (MM1) and J51 (VV2), lost infectivity at 134 °C. Boiling in 3% SDS alone reduced infectivity but did not eliminate it completely. However, when autoclaving at 121 or 134 °C was performed after SDS boiling, we could not detect the infectivity from all wires within the scope of our bioassay.

Regarding alkaline (NaOH) treatment, all sCJD strains retained infectivity after immersion in 1 M or 2 M NaOH aqueous solution at 20–25 °C for 1 h. When autoclaving at 121 °C was performed following alkaline immersion, infectivity was reduced compared to alkaline treatment alone. In particular, the H3 (MM1) infectivity was more significantly reduced at the higher concentration of 2 M NaOH, whereas no concentration-dependent effect was observed for Ak (VV2) and J51 (VV2). After additional autoclaving at 134 °C, no infectivity was detected in any of H3 (MM1), Ak (VV2) or J51 (VV2).

**Fig. 3.**
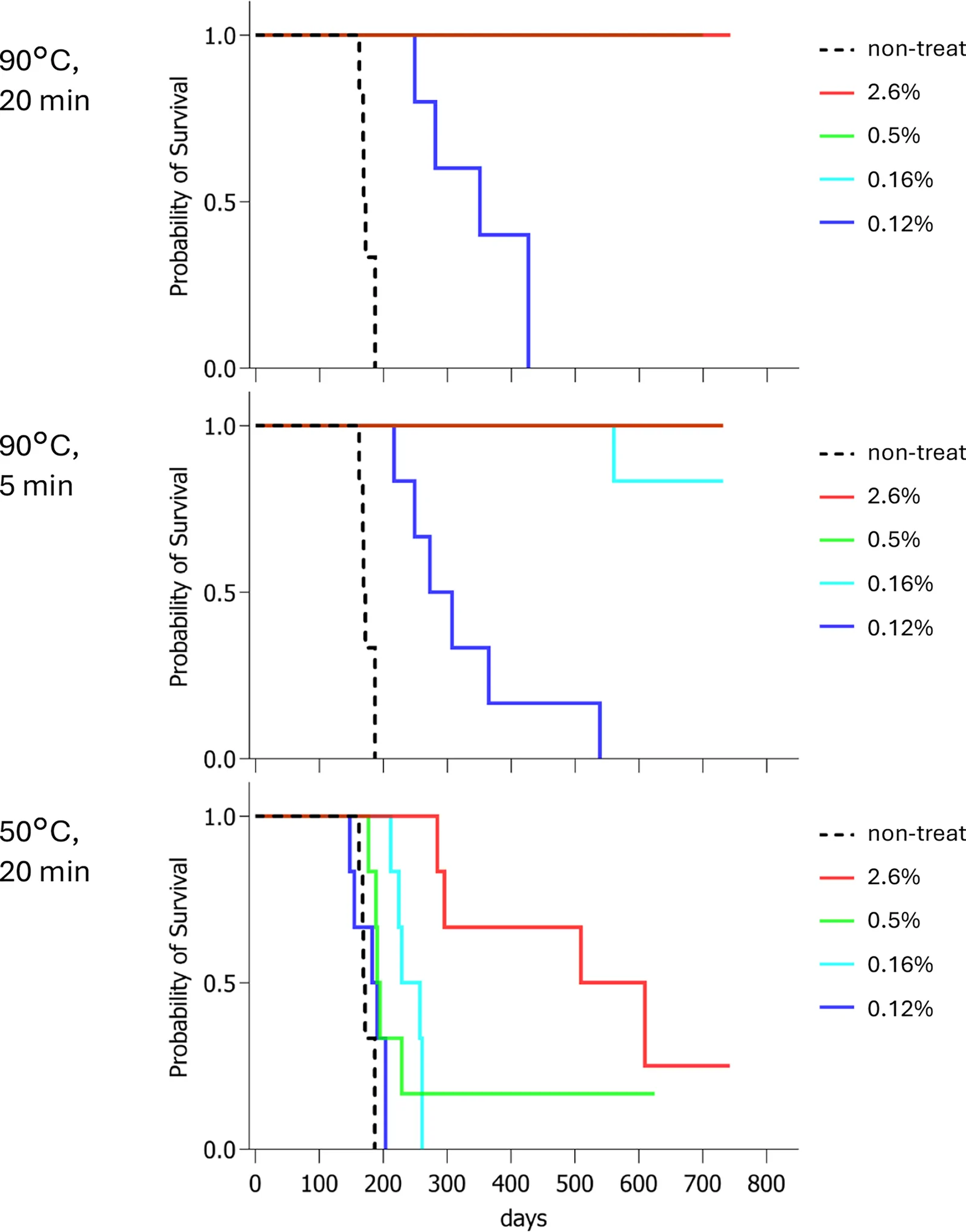
Inactivation of sCJD MM1 prion by an alkaline cleaner. Kaplan–Meier survival curves were generated to depict the probability of survival, reflecting incubation periods (days), in KiChM mice intracerebrally implanted with sCJD MM1-coated wires. Wires were treated with different concentrations of an alkaline cleaner (S Clean 55α: 2.6, 0.5, 0.16 or 0.12%) under various thermal conditions (90 °C for 20 min, 90 °C for 5 min or 50 °C for 20 min). Non-treated wires adsorbed with 10% brain homogenate were included as the ‘non-treat’ control. The overall log-rank test was significant (*P*<0.0001). Pairwise comparisons suggested trends towards differences; however, these were statistically non-significant after Holm adjustment for multiple testing, except for mild treatment (50 °C for 20 min). The different coloured lines correspond to each treatment condition and are shown on the right.

**Fig. 4.**
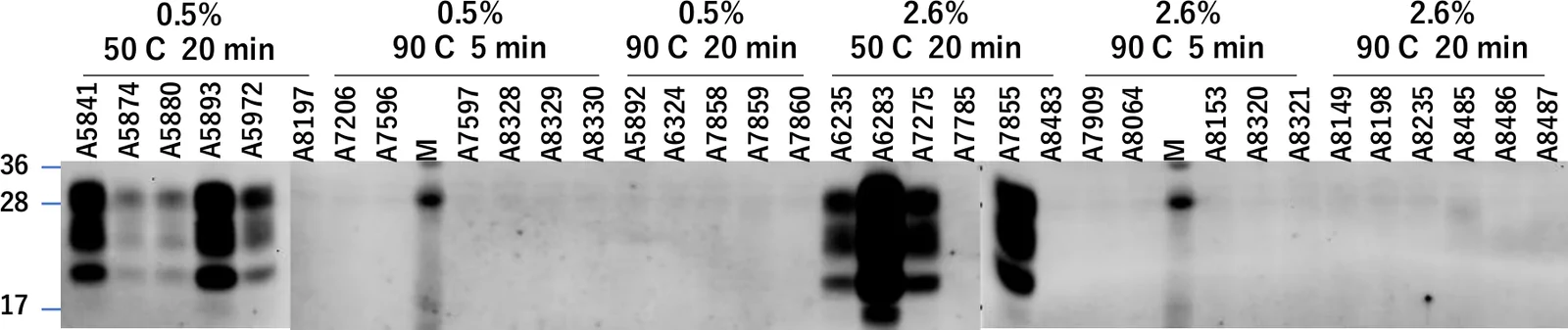
All of the diseased sacrificed and dead mouse brains were analysed by Western blotting to confirm prion transmission. Samples from two groups treated by 0.5 and 2.6% concentrations are shown. The numbers above the horizontal bars indicate the concentration of the alkaline cleaner and the treatment temperature and time. A number indicates the individual mouse number, and M indicates the molecular marker. Each lane was applied with 0.25 mg of brain sample. The antibody for prion-detecting antibody is anti-ChW (1:5000), for 4 °C overnight.

Additionally, inactivation tests were also conducted using an alkaline detergent – S CLEAN 55α – commonly used in washer-disinfector for surgical instruments in hospitals. These tests were performed only with H3 (MM1). The results are shown in Table 3, Fig. 3 and Fig 4.

**Table 3.** Infectivity after treatment with the alkaline cleaner on the sCJD-coated wire

	MM1 (H3)	
Inactivation procedure	n/n_0_*	Incubation period (mean days±sd)
**Alkaline cleaner (S CLEAN 55α)**		
2.6%, 90 °C, 20 min, pH 12.5	0/6	>743
2.6%, 90 °C, 5 min, pH 12.5	0/5	>732
2.6%, 50 °C, 20 min, pH 12.5	4/6	425±161
0.5%, 90 °C, 20 min, pH 11.8	0/5	>700
0.5%, 90 °C, 5 min, pH 11.8	0/6	>732
0.5%, 50 °C, 20 min, pH 11.8	5/6	196±20
0.16%, 90 °C, 20 min, pH 11.0	0/6	>700
0.16%, 90 °C, 5 min, pH 11.0	1/6	561
0.16%, 50 °C, 20 min, pH 11.0	6/6	241±22
0.12%, 90 °C, 20 min, pH 10.7	4/6	327±79
0.12%, 90 °C, 5 min, pH 10.7	6/6	325±117
0.12%, 50 °C, 20 min, pH 10.7	5/5	181±24

* Number of positive (n) and implanted mice (n_0_).

† Each wire is coated with 10% sCJD brain homogenate.

‘>number’ means the longest survival days of the mouse in the group.

The coated wires were immersed in S CLEAN 55α at high (2.6%, pH 12.5) to low (0.12%, pH 10.7) concentrations. When wires were treated at 90 °C for 5 or 20 min with 2.6 or 0.5% concentrations, these wire-coated M1 prions did not transmit infectivity to KiChM mice. However, when treated at 50 °C, infectivity was retained across all concentrations. Detergent concentrations of 0.16% (pH 11.0) or 0.12% (pH 10.7) did not eliminate infectivity to mice, except for the 0.16% concentration at 90 °C for 20 min (Fig. 4).

## Discussion

From the perspective of animal welfare, the number of mice per group at the beginning of the experiment was limited to five or six, and mice that died due to clear accidents early in the study were excluded from the statistical analysis. It is undeniable that this approach reduces statistical precision. To compensate for the limited group sizes in the V2 prion experiments, we utilized the VV2/J51 strain along with the VV2/Ak strain (Table 2).

The evaluation of prion sterilization methods using stainless steel wires coated with contaminated prions is widely regarded as the gold standard [18–24]. Wire implantation showed high sensitivity for detecting surface-bound infectivity, with detection limits comparable to, and in some cases higher than, direct inoculation of brain homogenate, demonstrating the risk of infection even with microscopic amounts of prion contamination on surgical instruments and other items (Supplementary Material 1).

WHO guidelines recommend autoclaving at 134 °C for 18 min as effective [25]. In this study, the M1 strain was hardly inactivated even after heating at 134 °C for 20 min, while the V2 strain of sCJD dried on a stainless steel wire surface was not detected as infectious (Table 2 and Fig. 2). Similar strain-specific differences in prions have been reported for scrapie and BSE [8, 9]. Regarding human prions, one report indicates that after autoclaving at 134 °C for 5 min, vCJD remains infectious, while VV2 becomes almost undetectable [11]. This report uses the PMCA method to inactivate the sCJD VV2 prion at 134 °C and does not mention the MM1 prion. Our experiment is the first to reveal a clear difference in heat resistance between the MM1 and VV2 strains of human sCJD prions. Furthermore, the inability to inactivate the sCJD MM1 prion even after autoclaving at 134 °C for 20 min highlights a significant limitation of current sterilization protocols.

Previous studies indicate that 93% of normal Japanese individuals have the 129 M/M genotype, and in Europe, an analysis of autopsy cases in a Dutch surveillance study reported that 75% of sCJD cases had M1 prions [1, 26]. Because the majority of sCJD cases carry M1 prions, the fact that they remain infectious after 20 min at 134 °C poses a major safety concern. While further confirmation using alternative methods is needed, these findings suggest that existing guidelines should be revised to ensure effective prion decontamination and minimize the risk of iatrogenic infection.

Neither the SDS boiling nor the NaOH soaking method alone achieved inactivation of M1 and V2 prions on the wires (Table 2 and Fig. 2). These findings are consistent with previous reports on SDS boiling for CJD mouse prions and NaOH soaking for prions from scrapie, sCJD VV2 or vCJD [27]. Notably, this study demonstrates that combining sterilization methods with different mechanisms can eliminate the practical infectivity of both MM1 and VV2 prions. In practice, the recommended approach is a 3% SDS treatment combined with autoclaving at 121 °C. However, even a simplified method – autoclaving in 3% SDS – showed residual infectivity in one of nine samples of Ak (VV2) after treatment at 121 °C. Only one positive mouse observed after a prolonged incubation period indicates a reduction in infectivity by ~10^−6^, which might be a sufficient effect to justify the use of this method as a simplified approach.

Our studies show NaOH soaking produced paradoxical results. sCJD MM1 infectivity is less reduced than that of sCJD VV2 after 1 M NaOH treatment, whereas MM1 was more reduced than VV2 after 2 M NaOH treatment, indicating the unreliability of using NaOH for prion inactivation. However, even combining NaOH and autoclaving at 121 °C was not effective for the inactivation of M1 and V2 prions, whereas combining NaOH and 134 °C autoclaving was sufficient. NaOH treatment followed by autoclaving at 134 °C eliminates infectivity of M1 prions. However, autoclaving at 134 °C alone also eliminates infectivity of V2, making the effectiveness of combining NaOH and autoclaving at 134 °C for VV2 questionable. Although the WHO recommends the combined use of NaOH treatment and autoclaving, our results suggest that the most effective combined treatment is SDS treatment followed by autoclaving at 121 °C.

Commercially available alkaline cleaner commonly used for cleaning and disinfecting instruments rendered M1 prions undetectable under essential conditions (above a certain concentration, processing temperature and pH) (Table 3 and Fig. 3). Due to space limitations in the animal housing facility, V2 prion testing was not performed, but M1 prions were inactivated under the essential conditions.

Bélondrade *et al*. compared steel wires contaminated with vCJD prions using the PMCA method and transgenic mice and tested commercially available cleaning agents currently used in hospitals with both protocols, reporting that most cleaning agents were ineffective against human prions [28]. Our findings in this study demonstrate that the concentration, temperature and pH of alkaline detergents play a crucial role in the cleaning of surgical instruments, which is essential for preventing secondary infections in M1 strain of sporadic sCJD.

In this study, we demonstrated that both M1 and V2 prions can be practically inactivated without relying on harsh conditions such as ultra-high-temperature autoclaving. This result was made possible by combining sterilization methods with different mechanisms. These findings suggest that it is possible to avoid secondary infections of M1 and V2 prion under the practical conditions by combining procedures that were previously considered insufficient when used alone. We propose a method for prion inactivation that combines common instrument sterilization processes that can be implemented at the hospital level, known as the ‘Two step processing method’.

In conclusion, preventing surgical contamination by human sporadic prions requires highly sensitive assay systems utilizing human prions. Sterilization methods evaluated using prions from other species may result in unnecessarily stringent conditions or insufficient protocols. This study employed sCJD, M1 and V2 prions, known to cause acquired prion disease, and provides critical and clinically relevant information.

## Supplementary material

10.1099/jgv.0.002326 Supplementary Material 1.

## Abbreviations

CJD: Creutzfeldt–Jakob disease
DDW: distilled and deionized water
iCJD: iatrogenic CJD
IHC: immunohistochemistry
PMCA: protein misfolding cyclic amplification
RT-QuIC: real-time quaking-induced conversion
sCJD: sporadic Creutzfeldt–Jakob disease
