# Supplementary material for: A two-step processing method is recommended to inactivate human sporadic M1 and V2 prions
Source: J Gen Virol. 2026 Sep 16;107(9):002326. doi: 10.1099/jgv.0.002326 (PMC13581090; doi:10.1099/jgv.0.002326)
Supplement: Supplementary file 1 [file jgv-107-02326-s001.pdf]

Supplementary 1

To clarify sCJD infectivity, the brain homogenates from MM1 (H3) or VV2 (Aki) were serially diluted 10-fold. From each dilution, 20 microliters were inoculated intracerebrally into three lines of humanised knock-in mice. The infection ratio (n/n0) and incubation period were defined as mentioned in the Methods section of this manuscript. Results are summarised in Table S1.

KiChM mice, which express a chimeric human/mouse prion protein, exhibited a shortened incubation period of 140 days following intracerebral inoculation with a 10^{-1} dilution of H3 (MM1), with the limit of detection (LOD) determined at the 10^{-6} dilution. KiMM mice, expressing human prion protein with the methionine homozygous genotype, showed an incubation period of 534 days at the 10^{-1} dilution of H3 (MM1), with the LOD at the 10^{-5} dilution. KiVV mice, expressing human prion protein with the valine homozygous genotype, had an incubation period of 319 days at the 10^{-1} dilution of Aki (VV2), and the LOD was determined at the 10^{-7} dilution, assuming that no mice were infected at the 10^{-8} dilution. Additionally, the estimated LD_{50} values were 7.07 $\log_{10}$ per gram of brain tissue for H3 (MM1) and 7.90 $\log_{10}$ per gram for Aki (VV2). These results indicate that KiChM and KiVV are suitable for detecting the infectivity of H3 (MM1) and Aki (VV2), respectively.

Table S1. Infectivity of sCJD brain homogenate of serial dilute in different mice

Dilution rate ($\log_{10}$)	MM1(H3) into KiChM		MM1 (H3) into KiMM		VV2 (AK) into KiVV	
	n/n_0‡	Incubation period (mean days ± SD)	n/n_0‡	Incubation period (mean days ± SD)	n/n_0‡	Incubation period (mean days ± SD)
-1¶	5/5	140 ± 8	4/4	534 ± 20	6/6	319 ± 13
-2	6/6	156 ± 7	5/5	541 ± 9	7/7	332 ± 10
-3	6/6	184 ± 22	5/5	617 ± 88	6/6	335 ± 21
-4	6/6	219 ± 21	5/5	656 ± 84	6/6	376 ± 28
-5	3/6	240 ± 65	1/5	712	5/5	441 ± 31
-6	1/6	777	0/5	>815	2/4	526, 622
-7	1/6	818	0/5	>815	1/5	474
-8	0/5	>840		Nt		Nt

‡ Number of positive(n) and inoculated mice (n_0).

¶ − 1 is 10% brain homogenate, and -2, -3,……show 10-fold dilution magnification each.

Nt: not tested.

“>number” means the longest survival days of the mouse in the group.

Supplementary 2

Figure S1

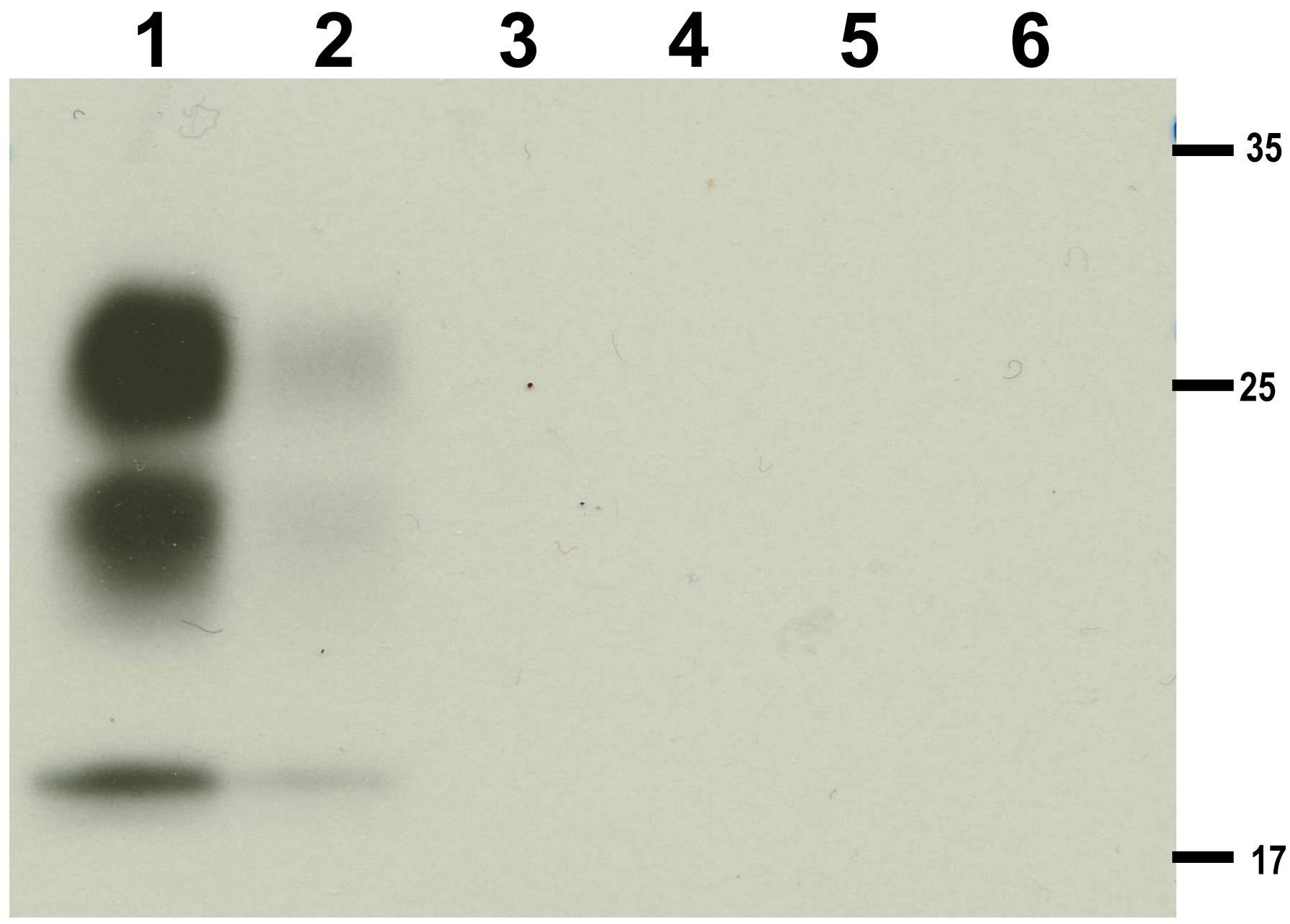

	1	2	3	4	5	6
IHC	P	Q	Q	N	N	N
WB	P	P	N	N	N	N
Final	+	+	-	-	-	-

Figure S1 legends

Evaluation methods of humanised mouse model in this study.

Lane 1~6: Individual KI-Hu129V/V mouse

IHC: immunohistochemistry: P; positive, Q; questionable, N; negative

WB; western blotting: P; positive, N; negative

Final; final evaluation: +; infected, -; non-infected

The numbers 35, 25, and 17 on the right represent the molecular weights (kD).
